# GEM-TREND: a web tool for gene expression data mining toward relevant network discovery

**DOI:** 10.1186/1471-2164-10-411

**Published:** 2009-09-03

**Authors:** Chunlai Feng, Michihiro Araki, Ryo Kunimoto, Akiko Tamon, Hiroki Makiguchi, Satoshi Niijima, Gozoh Tsujimoto, Yasushi Okuno

**Affiliations:** 1 Department of Systems Bioscience for Drug Discovery, Graduate School of Pharmaceutical Sciences, Kyoto University, 46-29 Yoshidashimoadachi-cho, Sakyo-ku, Kyoto 606-8501, Japan; 2 Kyoto University Education Unit for Global Leaders, 46-29 Yoshidashimoadachi-cho, Sakyo-ku, Kyoto 606-8501, Japan; 3 Bio Science Group, IT Solution Division 1, Industry Solution Business Unit, Mitsui Knowledge Industry, Osaka 531-0072, Japan; 4 Department of Genomic Drug Discovery Science, Graduate School of Pharmaceutical Sciences, Kyoto University, 46-29 Yoshidashimoadachi-cho, Sakyo-ku, Kyoto 606-8501, Japan

## Abstract

**Background:**

DNA microarray technology provides us with a first step toward the goal of uncovering gene functions on a genomic scale. In recent years, vast amounts of gene expression data have been collected, much of which are available in public databases, such as the Gene Expression Omnibus (GEO). To date, most researchers have been manually retrieving data from databases through web browsers using accession numbers (IDs) or keywords, but gene-expression patterns are not considered when retrieving such data. The Connectivity Map was recently introduced to compare gene expression data by introducing gene-expression signatures (represented by a set of genes with up- or down-regulated labels according to their biological states) and is available as a web tool for detecting similar gene-expression signatures from a limited data set (approximately 7,000 expression profiles representing 1,309 compounds). In order to support researchers to utilize the public gene expression data more effectively, we developed a web tool for finding similar gene expression data and generating its co-expression networks from a publicly available database.

**Results:**

GEM-TREND, a web tool for searching gene expression data, allows users to search data from GEO using gene-expression signatures or gene expression ratio data as a query and retrieve gene expression data by comparing gene-expression pattern between the query and GEO gene expression data. The comparison methods are based on the nonparametric, rank-based pattern matching approach of Lamb et al. (Science 2006) with the additional calculation of statistical significance. The web tool was tested using gene expression ratio data randomly extracted from the GEO and with in-house microarray data, respectively. The results validated the ability of GEM-TREND to retrieve gene expression entries biologically related to a query from GEO. For further analysis, a network visualization interface is also provided, whereby genes and gene annotations are dynamically linked to external data repositories.

**Conclusion:**

GEM-TREND was developed to retrieve gene expression data by comparing query gene-expression pattern with those of GEO gene expression data. It could be a very useful resource for finding similar gene expression profiles and constructing its gene co-expression networks from a publicly available database. GEM-TREND was designed to be user-friendly and is expected to support knowledge discovery. GEM-TREND is freely available at .

## Background

One of the major challenges in the post-genomic era is to understand how genes and their products interact to form functional networks. DNA microarray technology, which can simultaneously measure the expression of thousands of mRNAs, provides us with the first step toward the goal of uncovering gene functions on a genomic scale [1]. In recent years, vast amounts of gene expression data have been collected, much of which are available in public databases, such as the Gene Expression Omnibus (GEO) [2], ArrayExpress [3] and researchers' websites. These resources serve at least two purposes. One is as an archive of the data, which allows other researchers to confirm results that have been already published. A second use is to permit novel analyses of the data that go beyond what was envisioned or possible at the time of the original study [1,4-6]. However, to date, most researchers manually retrieve data from databases through web browsers using accession numbers (IDs) or keywords and switch to other tools for further analysis (e.g. network analysis), hence the need to continually import/export and reformat data [7,8]. The data retrieved using keywords or IDs is also usually limited by experimental conditions such as microarray platform, reagent, and cell type. In recent years, gene-expression patterns have been introduced as a new strategy to connect different biological states, and several methods were proposed to detect similarities among the gene-expression patterns of different biological states. Lamb et al. [9] introduced the Connectivity Map as a web tool to detect similar gene-expression signatures quantitatively among their original microarray dataset, which was observed under unified experimental conditions (the usage of cultured human cells treated with bioactive small molecules) by the specified laboratory teams. The other tools, L2L [10] and LOLA [11] have been also provided to compare users' data to published microarray data from different experimental conditions. As the similarity metrics, L2L and LOLA used the co-occurrence of genes (the number of overlap genes) between query gene list and pre-defined lists of differentially expressed genes compiled from published microarray data, and cannot measure gene-expression patterns quantitatively. Thus the existing mining tools allow users to search gene expression data from public databases, but these are also restricted by gene annotation, pre-selected gene lists, or experimental conditions. In order to detect similar gene-expression patterns across a public gene expression database, which consists of diverse data generated using different microarray platforms and by individual laboratory groups, we have developed a web tool named GEM-TREND (Gene Expression data Mining Toward Relevant Network Discovery) to automatically retrieve gene expression data across a wide range of microarray experiments in the publicly available GEO database by comparing gene-expression patterns between a query and the database entries. Subsequently, the system generates a gene co-expression network for retrieved gene expression data and may provide insights into unknown functional relationships of the genes.

## Implementation

GEM-TREND runs on a Linux server (Intel Xeon 2.8 GHz, 4G RAM). It combines a MySQL database management system (5.0.22) to store the pre-processed data with a dynamic web interface based on PHP (5.2.3). Data processing is performed using PHP and the R statistical package (2.5.1), and graphical representations are generated using a Java Applet graphical user interface. GEM-TREND provides both gene-expression pattern-based and text-based searches to retrieve gene expression data from GEO. For the former searches, the input data can be gene-expression signatures represented by a set of genes with up- or down-regulated labels or by gene expression ratio data. For text-based searches meanwhile, the input data can be keywords and accession IDs. Retrieved gene expression data can then be viewed as a co-expression network with gene ontology (GO) annotation, whereby genes and annotations are dynamically linked to external data repositories.

### Construction of reference gene expression profiles

The current system stores a wider spectrum of reference gene expression profiles compared to the Connectivity Map. In this study, the reference gene expression profiles were constructed as described below:

(1) Gene expression data annotated as treatment instances (i.e. treatment versus control) were extracted from the GEO database, amounting to 1540 GEO series and 41516 samples;

(2) For each sample, genes were ranked in descending order, according to the log ratio of treatment to control;

(3) Varying gene names/IDs dependent on microarray platforms were converted to UniGene IDs in accordance with the respective gene annotation files.

**Figure 1:**
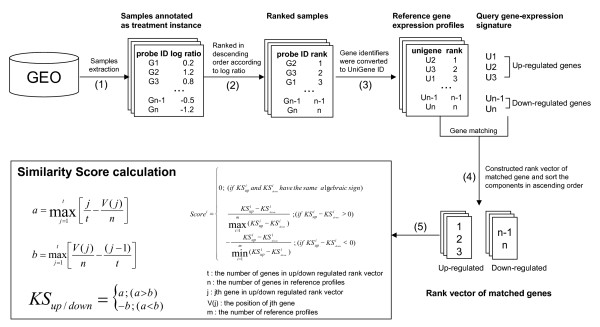
**The procedure of reference gene expression profiles construction and similarity score calculation**. (1) Gene expression data annotated as treatment instances (i.e. treatment versus control) were extracted from GEO. (2) For each sample, genes were ranked in descending order according to the log ratio of the treatment to control. (3) Varying gene identifiers (gene names/IDs) were converted to UniGene IDs according to the associated platform annotation file. (4) Constructed rank vector of up- and down-regulated genes that matched between the query and reference, respectively, and sort the components in ascending order. (5) Calculated similarity score.

These steps are schematically illustrated in Fig. 1. Samples lacking the associated annotation were filtered out, hence resulting in a total of 995 GEO series and 25974 samples. Table 1 summarizes the numbers of platforms, series and samples for each species. These samples were stored in a MySQL database as reference gene expression profiles.

**Table 1:** Distribution of rank-transformed data in GEM-TREND.

**Species**	**Number of platforms**	**Number of series**	**Number of samples**
*Homo sapiens*	207	444	12882
*Mus musculus*	89	250	6273
*Rattus norvegicus*	18	53	1654
*Arobidopsis thaliana*	8	16	161
*Drosophila melanogaster*	7	21	301
Others	69	211	4703

Total	398	995	25974

The data recently used as reference gene expression profiles in GEM-TREND.

### Gene expression data search

#### Gene-expression pattern-based search

GEM-TREND provides a gene-expression pattern-based search, by which we can explore reference gene expression data that resemble a given query in terms of pattern. The similarity is measured by the nonparametric, rank-based pattern matching approach of Lamb et al. [9]. In brief, Kolmogorov-Smirnov (KS) scores are calculated for both the up-regulated gene set (KS_up_) and down-regulated gene set (KS_down_) of the query, and these scores are integrated into a single score on the basis of the magnitude and signs of KS_up _and KS_down _(see Fig. 1 and Ref. [9] for the detailed calculation). Note that the gene expression profiles derived from multiple chips in the same experiment are counted as different hits. GEO samples corresponding to the reference profiles are then ranked in descending order of scores. Samples with larger positive scores are considered to be more closely correlated with the query, and vice versa.

GEM-TREND accepts a maximum of 500 genes as a query gene-expression signature. If the query is given in the form of gene expression ratio data, its signature will be automatically generated by GEM-TREND. Specifically, all genes are ranked in descending order according to their absolute ratio value, and genes exhibiting more than 2-fold change are selected from the 500 top-ranked genes. Finally, the selected genes are divided into up- and down-regulated sets according to the signs of their values.

**Figure 2:**
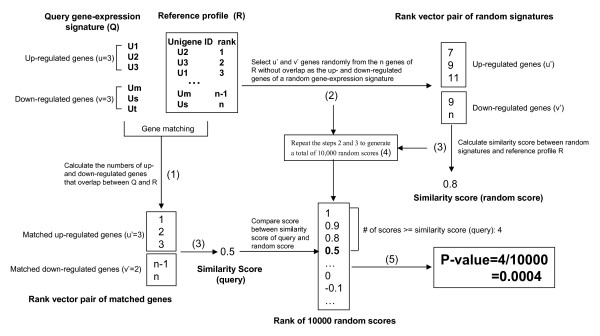
**The method for P-value calculation**. (1) Calculate the numbers of up- and down-regulated genes that overlap between Q and a reference profile R; let the numbers be u' (≤ u) and v' (≤ v), respectively. (2) Select u' and v' genes sequentially and randomly from the n genes of R without replacement, and construct a random signature; (3) Calculate the similarity score between R and the random signature; (4) Generated a total of 10,000 random scores by repeating steps 2 and 3. (5) The p-value associated with the similarity score (query score) between query Q and reference R is the proportion of random scores that are no less than the observed similarity score (query score).

In order to detect and reduce false negatives, we propose calculating the p-value associated with a similarity score using a randomization test. The procedure is as follows (Fig. 2):

(1) Given a query signature Q consisting of u up-regulated genes and v down-regulated genes, calculate the numbers of up- and down-regulated genes that overlap between Q and a reference profile R consisting of n genes; let the numbers be u' (≤u) and v' (≤v), respectively;

(2) Select u' and v' genes sequentially and randomly from the n genes of R without replacement, and construct a random signature;

(3) Calculate the similarity score (random score) between R and the random signature; 

(4) Repeat steps 2 and 3 to generate a total of 10,000 random scores.

(5) Estimate the p-value associated with the similarity score between Q and R, as the proportion of random scores that are no less than the observed similarity score.

#### Text-based search

GEM-TREND also allows users to search gene expression data by text (i.e. keywords, platform IDs, and series IDs). In this way, an N-gram based search engine is used, and GEO series title, series summary, platform IDs, and series IDs are considered as search criteria.

### Network generation and cluster analysis

In order to support delineating the relationship between genes, gene expression data retrieved by GEM-TREND is converted into a gene co-expression network that can identify the functionally related genes using GEO series data based on Pearson correlation coefficients and K-means clustering. First, the pairwise correlation coefficients are calculated for genes with more than 2-fold changes in expression levels, whereupon these genes are then clustered into N clusters using K-means clustering. N is determined using the DB index [12]. The cluster number with the largest DB is considered as N. Each gene represents a node and is connected to all the other genes in the same cluster based on correlation coefficients, hence sub-networks corresponding to clusters are generated and subsequently inter-connected, based on the Euclidean distance between them. Thus, the network was constructed. To reduce false positive links and to keep the graph size reasonable, the threshold of correlation coefficients and Euclidean distance is set to 0.92. Furthermore, each gene that appears on the network is annotated based on the associated GO term [13].

## Results and Discussion

### Overview of GEM-TREND

**Figure 3:**
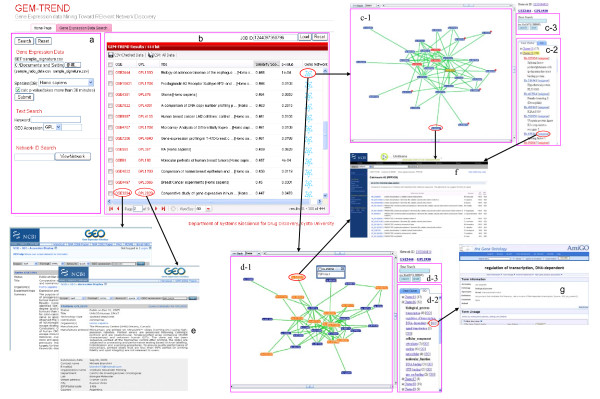
**Screenshot of GEM-TREND**. a) Query input area. The gene-expression signature, gene expression ratio data and text are accepted. Network IDs can be used to retrieve previous networks. b) Results area. The search results of GEO series ID (GSE ID), GEO platform ID (GPL ID), series title, similarity score, and p-value are displayed. One record corresponds to one GEO series and links to GEO by GSE ID and GPL ID. The previous results can be retrieved by JOB IDs. c) Network visualization (Gene Cluster tab): c-1) Network graphical display area. Genes (nodes) in red background are genes from query, while the genes in the yellow background are those that are user-selected. The number shown in the top-right of the genes describes the number of hidden linkages. These linkages can be expanded or hidden by a right click on the gene of interest to choose from the pop-up menu. Genes link to the UniGene database by double clicking. c-2) Gene cluster area, whereupon gene clusters are shown. The number following the cluster describes the number of member genes in the cluster. Genes link to the UniGene database by clicking the UniGene icon. c-3) Gene search window. Matched genes will be highlighted in the gene cluster area. d) Network visualization (GO tab): d-1) Network graphical display area. Genes in the orange background are those associated with the common GO term. d-2) Gene annotation. The top three significant shared GO terms of genes in each ontology are shown for each cluster. The number following the term describes the number of genes associated with the term. Terms link to GO by clicking the GO icon. d-3) Gene search window. e) Linkout to GEO database. f) Linkout to Unigene database. g) Linkout to Gene Ontology database.

GEM-TREND is designed to be user-friendly. Only a few simple steps are required to search GEO gene expression data and visualize the network. The main page of GEO gene expression data search comprises a query input area (Fig. 3a), and a results area (Fig. 3b). For a GEO gene expression data search, both gene-expression pattern-based searches (either gene-expression signatures or gene expression ratio data as inputs) and text-based searches (accepting keywords, platform IDs, or series IDs as inputs) are available, but similarity scores and p-values are calculated only for gene-expression pattern-based searches. To further analyze retrieved data (e.g. network analysis), GEM-TREND provides the GEO series that links together a group of related samples instead of providing reference gene expression profiles. The results consist of GEO series ID (GSE ID), GEO platform ID (GPL ID), series title, similarity score, and p-value displayed in the results area. Here, the similarity score of the GEO series is the maximum similarity score among samples in the same GEO series. The full series title can be displayed as a tool-tip when the mouse is over the title, and each series links to GEO by clicking the GSE ID or GPL ID (Fig. 3e). In addition, the series of interest can be selected for further processing. Both search results and selected series can be downloaded in CSV format.

Genes in each series can be viewed as a co-expression network by clicking the network icon (Fig. 3b). The network with GO annotation is shown on the network visualization page that comprises three major parts: the network graphical display area (Figs. 3c-1, 3d-1), the cluster information area (Figs. 3c-2, 3d-2), and the gene search window (Figs. 3c-3, 3d-3). The network graphical display area dynamically shows the full or sub-network according to the user's operation. On the network, genes from the query are highlighted, and the gene name is displayed as a tool-tip when the mouse is over the node (gene ID). The neighbor nodes (genes) can be expanded or hidden by right clicking on the node of interest to bring up a pop-up menu. In the cluster information area, clusters including their member genes are shown under the Gene Cluster tab. Users can click the Cluster Name to view the sub-network which includes co-expression genes, and click the UniGene ID to access the UniGene database [14] (Fig. 3f). Under the GO tab, the top three significant shared GO terms of genes in each of the ontologies (cellular component, biological process, molecular function) are shown for each cluster (Fig. 3d-2). The genes will be highlighted on the displayed network once the common function or process they perform is selected, and they also have a link to GO [13] (Fig. 3g). In addition, users can search for a gene of interest in the network using an ID or gene name through the gene search window. GEM-TREND also allows users to retrieve the previous results of both GEO data searches and network visualizations using the JOB ID and the network ID (the IDs are valid for two weeks) (See additional file 1-PDF-User guide for GEM-TREND for example).

### Validation of GEM-TREND

**Figure 4:**
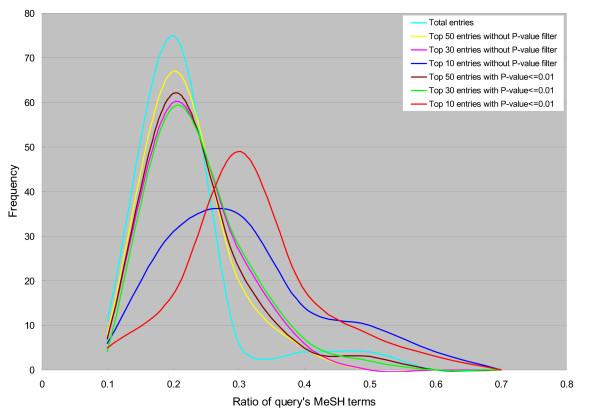
**The distribution of the ratio of the query's MeSH terms in the top-ranked entries for 100 randomly selected queries**. The groups in different color are the top 50 entries without a P-value filter, top 50 entries with a P-value <= 0.01, top 30 entries without a P-value filter, top 30 entries with a P-value <= 0.01, top 10 entries without a P-value filter, top 10 entries with a P-value <= 0.01, and total entries, respectively. The total entries represent all human species microarray series (corresponding to 444 series).

To validate whether GEM-TREND could retrieve the gene expression entries biologically related to a query, we evaluated the similarity of biological annotations between the query and the retrieved microarray data by using their MeSH terms. As a biomedical vocabulary thesaurus, the MeSH Term [15] is used by the National Library of Medicine (NLM) to index articles for the MEDLINE/PubMed database [16]. NCBI's Entrez link system [17] connects GEO data with related literature in PubMed. Hereby we assigned biological annotations in MeSH terminology to each entry of GEO microarray data via related literature, and we can estimate the biological relationship between a query and its retrieved data using their expression patterns. The validation was carried out with a set of 100 human species samples (gene expression ratio data) randomly extracted from GEO as queries as follows. First, for each query, GEM-TREND results in a ranking list of the gene expression data (GEO series) with their similarity score and P-value were estimated by the gene-expression patterns. Subsequently, we calculated the constituent ratio of the query's MeSH terms to those of the top ranked expression data on the selected criterion (top 10, 30 or 50 rank, and with or without the P-value). Fig. 4 shows the distribution of the ratio of a query's MeSH terms in the retrieved top-ranked entries. The distribution was generated by the retrievals of 100 randomly selected queries. As shown in Fig. 4, the peaks shifted to the right in the order of total, top 50, 30 and 10 entries. Importantly, filtering by P-value enabled the ratio of query's MeSH terms contained in the top ranked dataset to be increased more efficiently, indicating that our implemented P-value score is available to promote more effective exploration. These results demonstrate that GEM-TREND could retrieve biologically relevant microarray data across a wide range of microarray experiments in GEO by detecting the similarity of gene-expression pattern.

**Figure 5:**
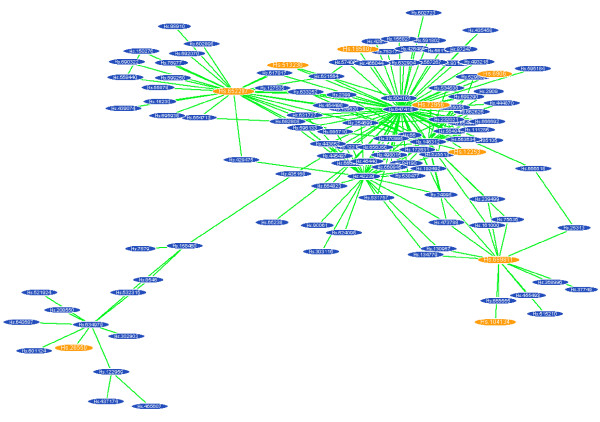
**A co-expression network generated using GSE1827 data**. The yellow-colored genes are categorized as GO0003700: transcription factor activity. Interestingly, these are hub genes or the neighbor genes in the sub-network, suggesting that the transcriptional factors might be key molecules for bladder tumors.

For further validation, we next used three types of in-house microarray data, which we previously reported but did not deposit in GEO, as the query examples: query-1) microarray data of human bladder cancer (Additional file 2-CSV-Gene expression profile of human bladder cancer) [18]; query-2) microarray data of rat chemical hepatocarcinogenesis (Additional file 3-CSV-Gene expression profile of rat chemical hepatocarcinogenesis)[19]; and query-3) microarray data of mouse mast cells pooled from stomach subregions (Additional file 4-CSV-Gene expression profile of mast cells pooled from mouse stomach subregions) [20]. In the score-ordered results of query-1 (P-value < 0.01), GSE1827 (titled "Waldman Bladder tumors") was ranked in fourth. Moreover, the top 10 entries showed appropriate annotations related to tumors, inflammatory and immune responses (Table 2). For the query-2 (P-value < 0.01), all among the top 5 entries were related to chemical-treated experiments, and seven entries among the top 10 were observed using rat liver samples (Table 3). The biological relationships among the top 10 results of query-3 (P-value < 0.02) were not clear, but GSE6192 (titled "Gene expression changes during murine mucosal mast cell in vitro differentiation") was found out in the twelfth rank (Table 4). These findings indicate the general applicability of GEM-TREND to external microarray queries independent of GEO. For further analysis, we generated gene co-expression networks from GSE1827 (a series of GEO microarray data) which was one of query-1 results (Fig. 5). GEM-TREND can provide us with the bladder tumor-associated networks from the query-1 consisting only the two DNA Chips. Note that in general a number of congeneric microarray data are required to construct gene co-expression networks. Thus, GEM-TREND can help comprehensive re-analysis of the primary data by merging data from multiple studies and provide insights into unknown functional relationships of the genes.

**Table 2:** Results of search using the gene expression profile of human bladder cancer - top 10 entries sorted by similarity scores with lowest P-value < 0.01.

**Series**	**Platform**	**Description**	**Similarity score**	**P-value**
GSE6112	GPL4475	Tubercolosis and healthy infected patients PBMC_TB_vs_Pool_LTBI	1	0.0054
GSE3901	GPL3279	Response of quiescent human fibroblasts to different growth factors and serum	0.96	0.0004
GSE1726	GPL564	Rosetta_Merck_CEPH_Study	0.904	0.0012
GSE1827	GPL1479	Waldman Bladder tumors	0.881	0.0072
GSE2845	GPL2567	Human breast tumor	0.854	0.0063
GSE60	GPL174	Diffuse large B-cell lymphoma	0.851	0.0029
GSE838	GPL564	Individual-specific variation of gene expression in peripheral blood leukocytes	0.845	0.0057
GSE3176	GPL1528	p53 In Inflamatory Stress Response	0.815	0.0001
GSE344	GPL273	Spotted long oligonucleotide arrays	0.813	0.0097
GSE7965	GPL3991	Blood and Adipose tissue samples	0.805	1.50E-03

The results were retrieved from human species reference profiles.

**Table 3:** Results of search using the gene expression profile of rat chemical hepatocarcinogenesis - top 10 entries sorted by similarity scores with lowest P-value < 0.01.

**Series**	**Platform**	**Description**	**Similarity score**	**P-value**
GSE5337	GPL890	Gene Expression Profiling In Rat Smooth Muscle Cells Modulated by Rapamycin and Paclitaxel.	1	1.00E-04
GSE5860	GPL890	Gene expression analysis of rat livers after exposure to acetaminophen	0.983	1.00E-04
GSE5652	GPL890	Blood and liver exposed to acetaminophen (APAP)	0.904	1.00E-04
GSE5595	GPL890	Acetaminophen (APAP) Rat Liver Test Gene Expression Data Set	0.895	1.00E-04
GSE5381	GPL890	Gene expression analysis of liver and kidney following methapyrilene treatment in male Sprague-Dawley rats	0.685	0.0015
GSE791	GPL542	GH inj old liver (1-7)	0.657	5.00E-04
GSE4270	GPL890	Aging Induced Alterations in Hepatic Gene Expression of the Male Fisher Rat	0.637	0.0045
GSE3608	GPL3076	Renal medullary genes in salt-sensitive hypertension	0.634	1.00E-04
GSE7483	GPL890	Microarray analysis of aortic tissue from different rat hypertensive models	0.634	0.0039
GSE789	GPL542	Effect of age liver	0.627	8.00E-04

The results were retrieved from rat species reference profiles.

**Table 4:** Results of search using the gene expression profile of mast cells pooled from mouse stomach subregions - top 20 entries sorted by similarity scores with lowest P-value < 0.02.

**Series**	**Platform**	**Description**	**Similarity score**	**P-value**
GSE3088	GPL2510	Expression profiling of Muscle tissue from (C57BL/6J × C3H/HeJ)F2 mice on ApoE null backgrounds	1	1.00E-04
GSE2814	GPL2510	Expression profiling of liver tissue from (C57BL/6J × C3H/HeJ)F2 mice on ApoE null backgrounds	0.881	4.50E-03
GSE4664	GPL891	24 hour time course: regulation of uterine genes by estradiol in ovariectomized mice	0.874	5.00E-04
GSE8104	GPL5137	Primary macrophage response to L. monocytogenes and bacteria-derived ligands	0.855	1.70E-03
GSE8100	GPL5137	WT and myd88-/- macrophage response to WT and hly- L. monocytogenes	0.847	6.00E-04
GSE2220	GPL1832	Genetic variation of gene expression is tissue specific in inbred mice	0.795	1.00E-02
GSE4615	GPL891	Estren Behaves as a Weak Estrogen Rather than a Non-genomic Selective Activator in the Mouse Uterus	0.792	0.0034
GSE7029	GPL2510	Zfp90 Transgenic Signature in Mouse White Adipose Tissue	0.788	2.50E-03
GSE7615	GPL2884	Cancer Process Study	0.752	0.0107
GSE7600	GPL2884	Atm-/-, mTerc-/-, p53-/- triple knock-out lymphoma vs normal mouse DNA (GPL2884)	0.752	1.13E-02
GSE3086	GPL2510	Expression profiling of Adipose tissue from (C57BL/6J × C3H/HeJ)F2 mice on ApoE null backgrounds	0.726	0.009
GSE6192	GPL891	Gene expression changes during murine mucosal mast cell in vitro differentiation	0.709	1.76E-02
GSE4248	GPL891	Identification of genes regulated by RORg in mouse thymus	0.705	1.99E-02
GSE3087	GPL2510	Expression profiling of brain tissue from (C57BL/6J × C3H/HeJ)F2 mice on ApoE null backgrounds	0.69	1.62E-02
GSE3516	GPL870	22k v1.0 EMvPL	0.605	0.0065
GSE3507	GPL870	FlAmp RNA Input Reduction	0.605	8.70E-03
GSE1013	GPL967	Gene Expression Profile of NHE1 Null Mutation	0.552	0.0134
GSE8625	GPL5530	Comparison of undifferentiated ES cell lines HM1, IMT11, SHBL6.3	0.488	1.82E-02
GSE8528	GPL5369	Expression analysis of gene differentially expressed in the developping ovary	0.455	0.0011
GSE3289	GPL2828	Chronic hypoxia alters the level, maturation and control of gene expression in mouse kidney	0.415	3.00E-03

The results were retrieved from mouse species reference profiles.

GEM-TREND can be considered as an extension of the Connectivity Map and a supplementary tool of GEO. Compared to the other microarray comparison tools such as L2L and LOLA, GEM-TREND has unique features in data resources, search method and main focus. The existing web tools use the pre-annotated lists of the limited genes (only a fraction of all available microarray data) as reference data, while GEM-TREND directly calculates complete raw data from GEO microarray resource, suggesting that GEM-TREND has an ability to access a greater number of public raw data. For the search method, the existing tools compare microarray data only by calculating the number of overlap genes, but GEM-TREND uses gene-expression pattern matching algorithm based on the nonparametric, rank correlation statistics. Moreover, compared to the existing tools which interpret new data using biologically significant genes annotated with published information, GEM-TREND focuses on data retrieval from GEO and gene-network analyses using GO annotation. GEM-TREND would thus be a unique and useful web tool to help researchers utilize GEO database more effectively, and to support knowledge discovery.

## Conclusion

GEM-TREND was developed to retrieve gene expression data by comparing the gene-expression pattern of queries with those of gene expression data in a public database based on the nonparametric, rank-based pattern matching approach with the additional calculation of statistical significance and to provide network visualization. It could be a very useful resource for finding similar gene expression profiles in an available public database and generating the associated co-expression networks. GEM-TREND was designed to be user-friendly and is expected to support knowledge discovery by providing a new means of data retrieval.

In future, the reference data will be automatically updated from GEO and other public databases. We also intend to find other appropriate ways to solve the limitations of false negatives caused by missing UniGene IDs and improve search speed.

## Availability and requirements

Project name: GEM-TREND

Project home page: 

Operating system: platform independent

Programming language: Java, PHP

Other requirements: Java 1.5.0 or higher

License: The tool is available free of charge

Any restrictions to of use by non-academics: None

## List of abbreviations

GEO: Gene Expression Omnibus; GO: Gene Ontology; GSE: Series in GEO; GPL: Platform in GEO; MeSH: Medical Subject Headings.

## Authors' contributions

CF designed the system and wrote the manuscript; MA gave comments and edited the manuscript; RK acquired GEO data; AT wrote the code; HM wrote the code and rank-transformed expression data; SN gave comments and performed test of system; GT led the project; YO led the project and edited the manuscript. All authors have read and approved the final manuscript.

## Supplementary Material

Additional file 1 **User guide for GEM-TREND**. User guide for GEM-TREND as a PDF file. Click here for file

Additional file 2 **Gene expression profile of human bladder cancer**. Gene expression profile of human bladder cancer presented as an Excel CSV file. Click here for file

Additional file 3 **Gene expression profile of rat chemical hepatocarcinogenesis**. Gene expression profile of rat chemical hepatocarcinogenesis presented as an Excel CSV file. Click here for file

Additional file 4 **Gene expression profile of mast cells pooled from mouse stomach subregions**. Gene expression profile of mast cells pooled from mouse stomach subregions presented as an Excel CSV file. Click here for file
