# Supplementary material for: GEM-TREND: a web tool for gene expression data mining toward relevant network discovery
Source: BMC Genomics. 2009 Sep 3;10:411. doi: 10.1186/1471-2164-10-411 (PMC2748096; doi:10.1186/1471-2164-10-411)

User guide for GEM-TREND

1. Requirements for Using GEM-TREND

GEM-TREND is implemented as a java applet which can be run in most common browsers and has been test with Internet Explorer 7.0, Internet Explorer 8.0, FireFox 2.0, Safari 3.2, Google Chrome 2.0 on different platforms. In some rare cases, the user may require to install JRE (Java Run-Time Environment) to run the GEM-TREND. JRE can be downloaded from SUN freely: http://www.java.com/ja/download/

2. Overview

GEM-TREND (Gene Expression data Mining Toward RElevant Network Discovery) is a web-based tool for retrieving gene expression data from Gene Expression Omnibus (GEO) by comparing gene-expression pattern of queries with those of GEO gene expression data and providing network visualization. The comparison methods are based on the nonparametric, rank-based pattern matching approach of Lamb et al. (Science 2006) with the additional calculation of statistical significance. GEM-TREND allows users find similar gene expression profiles without using keywords or IDs, and the results retrieved are not restricted by experimental conditions.

Retrieved gene expression data can then be viewed as a co-expression network with gene ontology (GO) annotation where genes and annotations are dynamically linked to external data repositories. The network analysis could provide insights into unknown functional relationships of the genes.

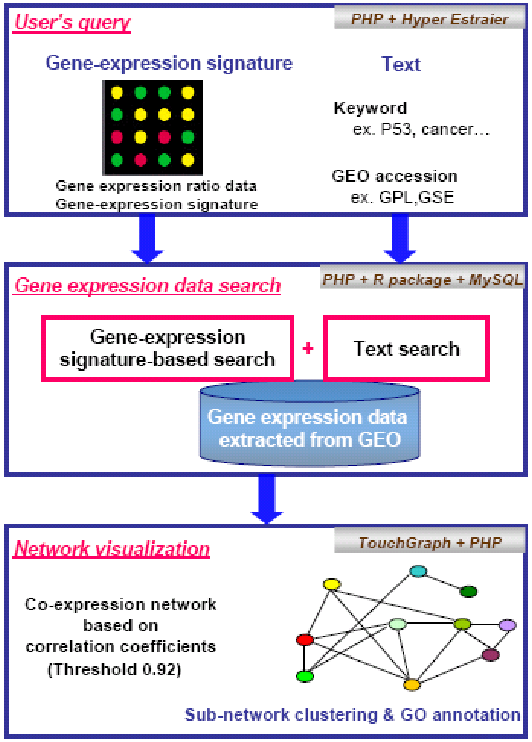

Figure 1. workflow of GEM-TREND

3. Gene expression data search

GEM-TREND provides both gene-expression pattern-based and text-based searches to retrieve gene expression data from GEO.

3.1 Gene-expression pattern-based search

3.1.1 Query preparing

For gene-expression pattern-based searches, the input data can be gene-expression signatures represented by a set of genes with up- or down-regulated labels or by gene expression ratio data. The format of the input data are shown as follows:

Format of gene-expression signature

Query of gene-expression signature is composed of two columns:
1) Gene identifier. This is the unique identifier for the gene. Recently, UniGene ID AffyMetrix probe ID and Agilent Probe ID are accepted.
2)Gene label: assign 1 for up-regulation gene, -1 for down-regulation gene.

Note: GEM-TREND accepts maxinium of 500 genes as signature.
Example is available at http://cgs.pharm.kyoto-u.ac.jp/services/network/sample/sample_signature.csv

Format of gene expression ratio data

Query of gene expression ratio data is composed of following two columns:
1)Gene identifier. This is the unique identifier for the gene. Recently, UniGene ID, AffyMetrix probe ID and Agilent Probe ID are accepted.
2)Log ratio of treatment to control:

Note: The number of genes is not limited, but only the top 500 differentially expressed genes will be automatically selected for search.
Example is available at http://cgs.pharm.kyoto-u.ac.jp/services/network/sample/sample_ratio_data.csv

3.1.2 Searching data using Gene-expression pattern (1) – Search –

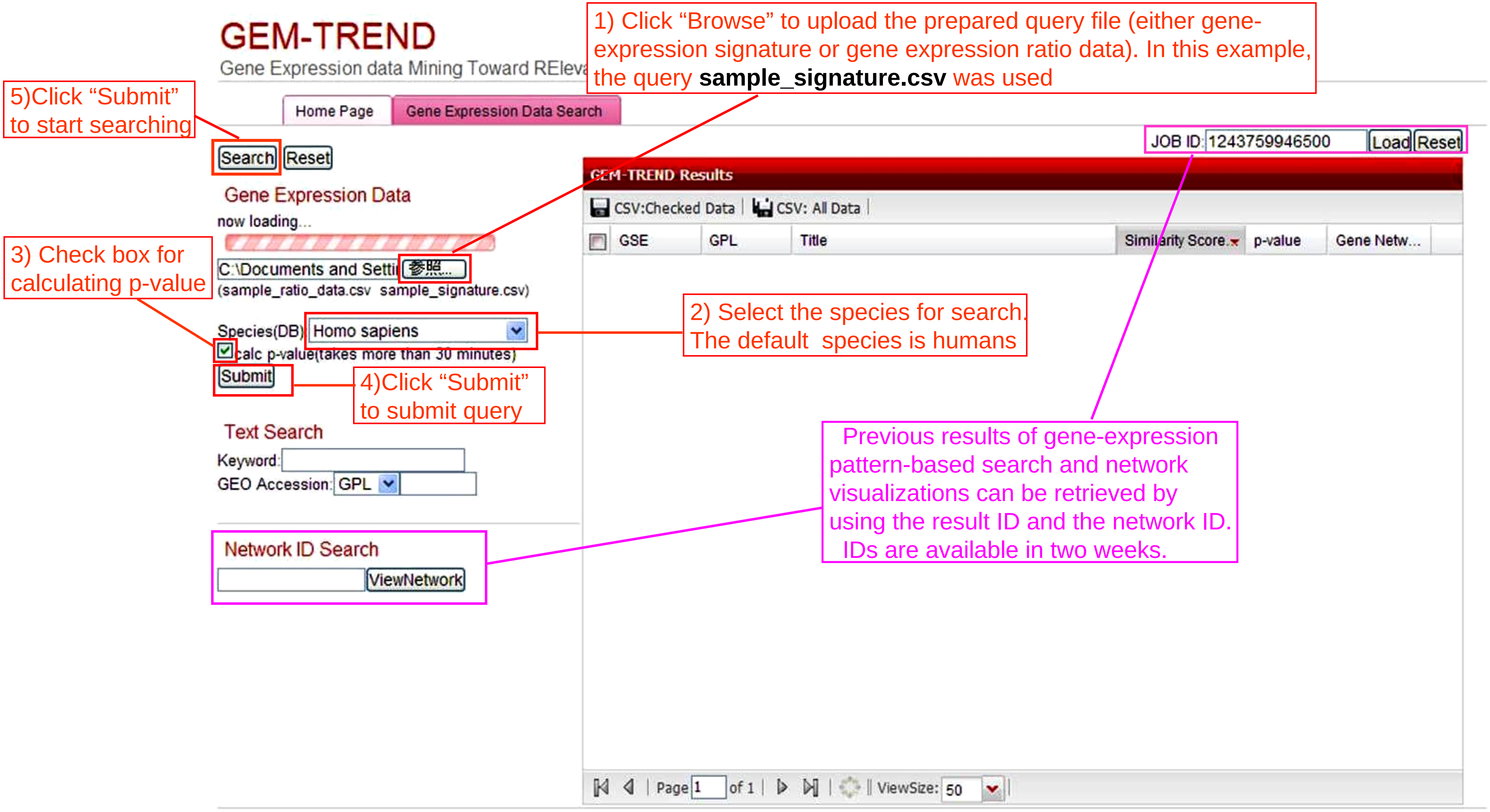

3.1.2 Searching data using Gene-expression pattern (2) –Result–

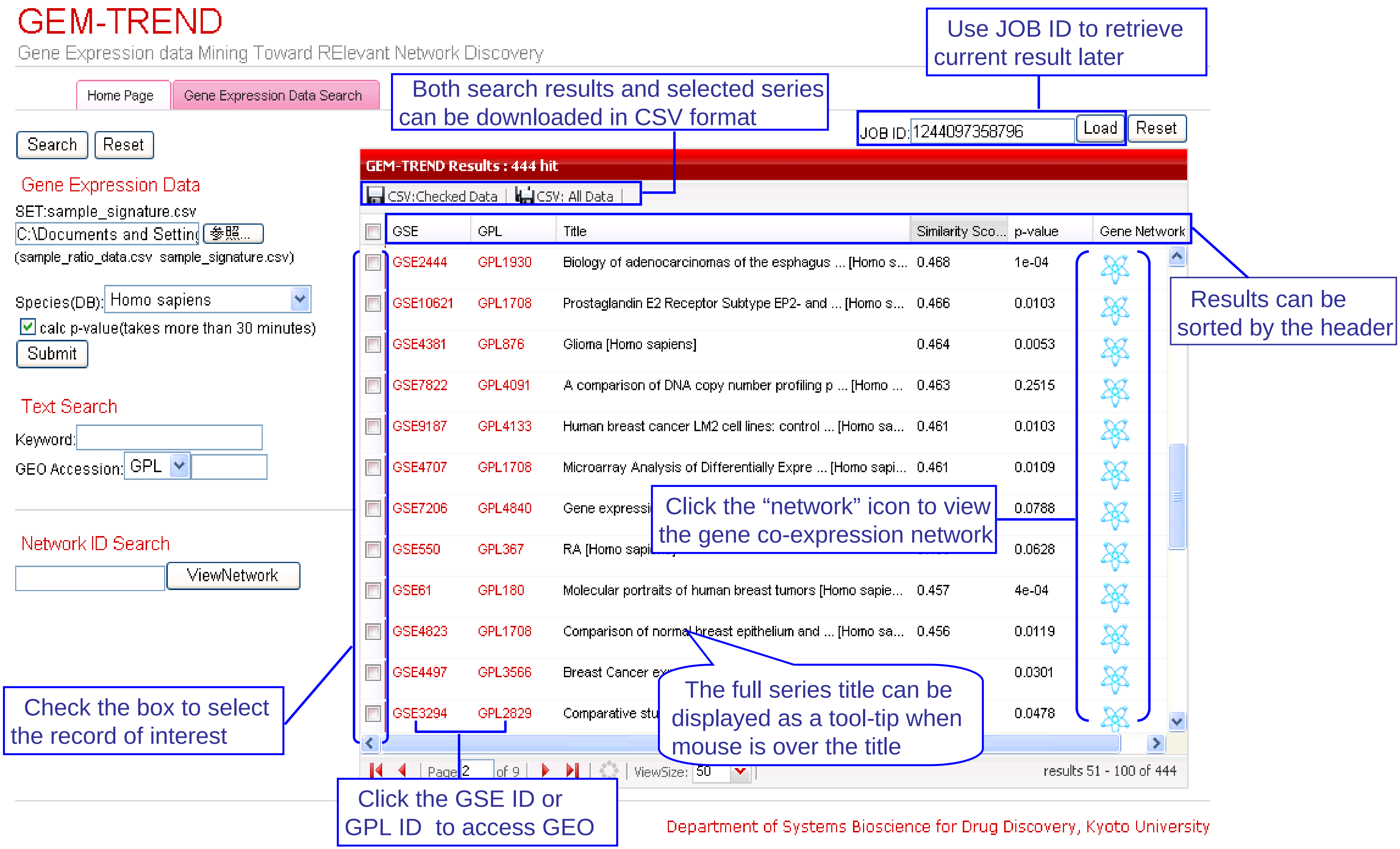

3.2 Text-based search

GEM-TREND accepts keywords (e.g. p53), GEO series ID, GEO platform ID as query. Text-based search can be used to directly retrieved data from GEO, or used as further search to retrieve data from the results of gene-expression pattern-based search.

3.2.1 Retrieve data from the results of gene-expression pattern-based search

For text-based search, the results ID is not available

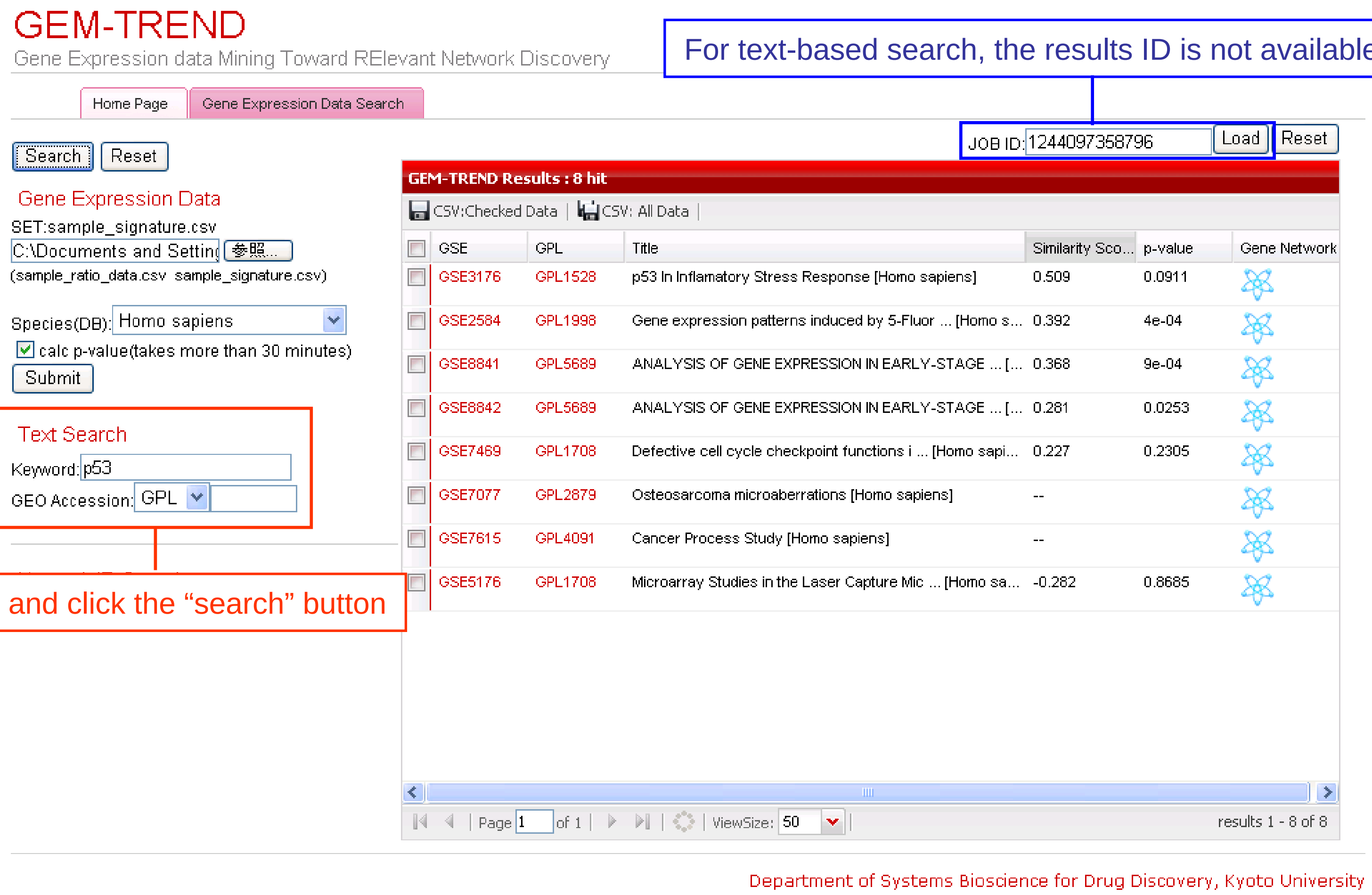

Input the keywords and click the “search” button

If both keywords and ID are inputted, the “AND search” will be performed.

3.2.2 Retrieve data by directly searching GEO

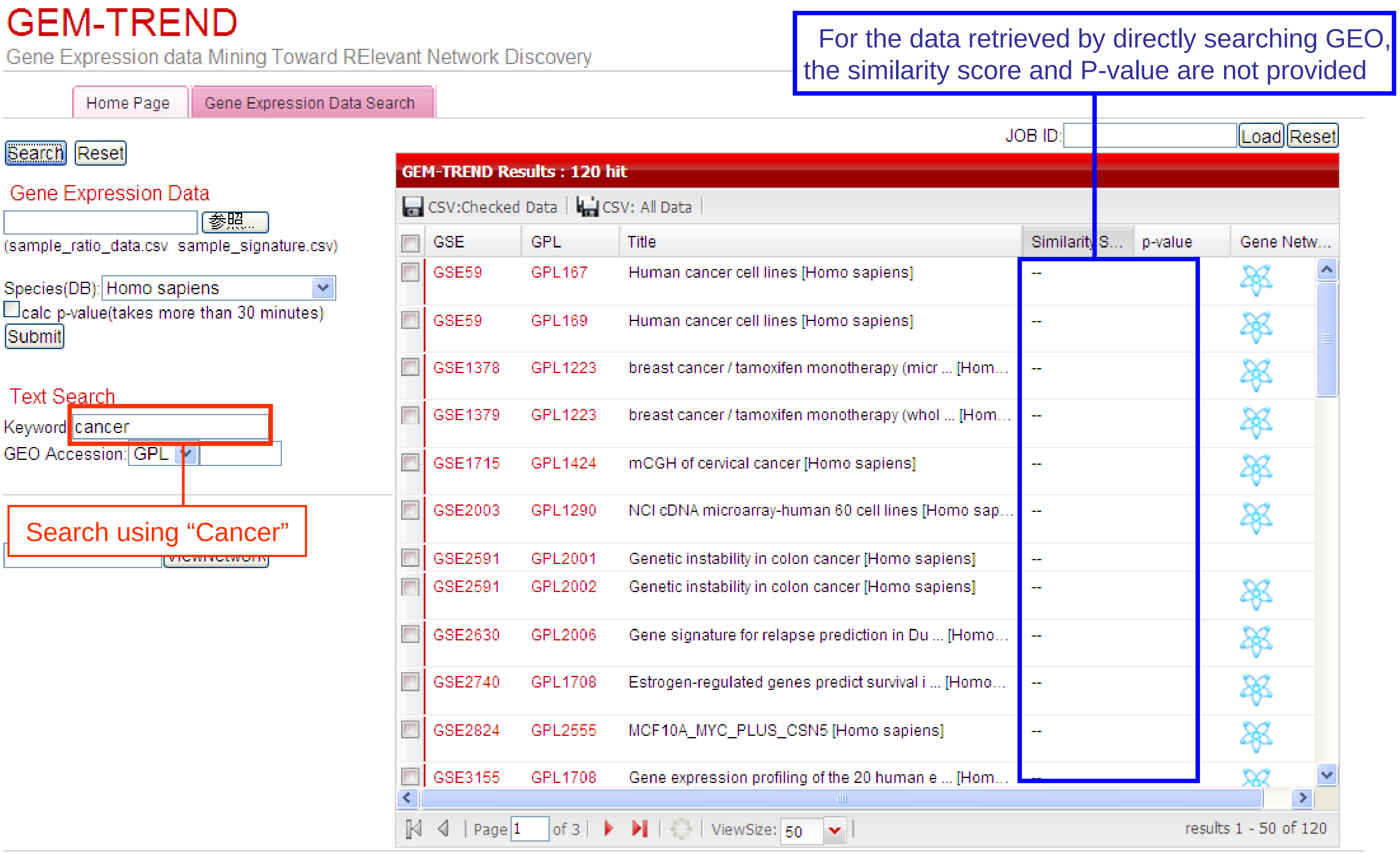

4. Network visualization

Retrieved gene expression data can be viewed as a co-expression network with gene ontology (GO) annotation where genes and annotations are dynamically linked to external data repositories. The network visualization page is composed of three major parts: the network graphical display area, the cluster information area, and the gene search window as showed in following figures. The network graphical display area dynamically shows the full or sub-network according to the user's operation.

Mouse Manipulation at the network graphical display area

- Left single mouse click will select the node. The selected node centered sub-network will be displayed.
- Right single mouse clicking will invoke the pop-out menu. It allows user to expand the node, and hid the links.
- Move the mouse over the node will show the annotation information of the node as shown in the above figure.
- Mouse dragging changes network style.
- On the top of graph panel, there are two modes can be used to change network size (zoom mode) and to rotate network (rotate mode).
- The network will be refleshed after new operation. The network needs to be expanded by right click to invoke the pop-out menu.

4.1 Network with gene cluster information

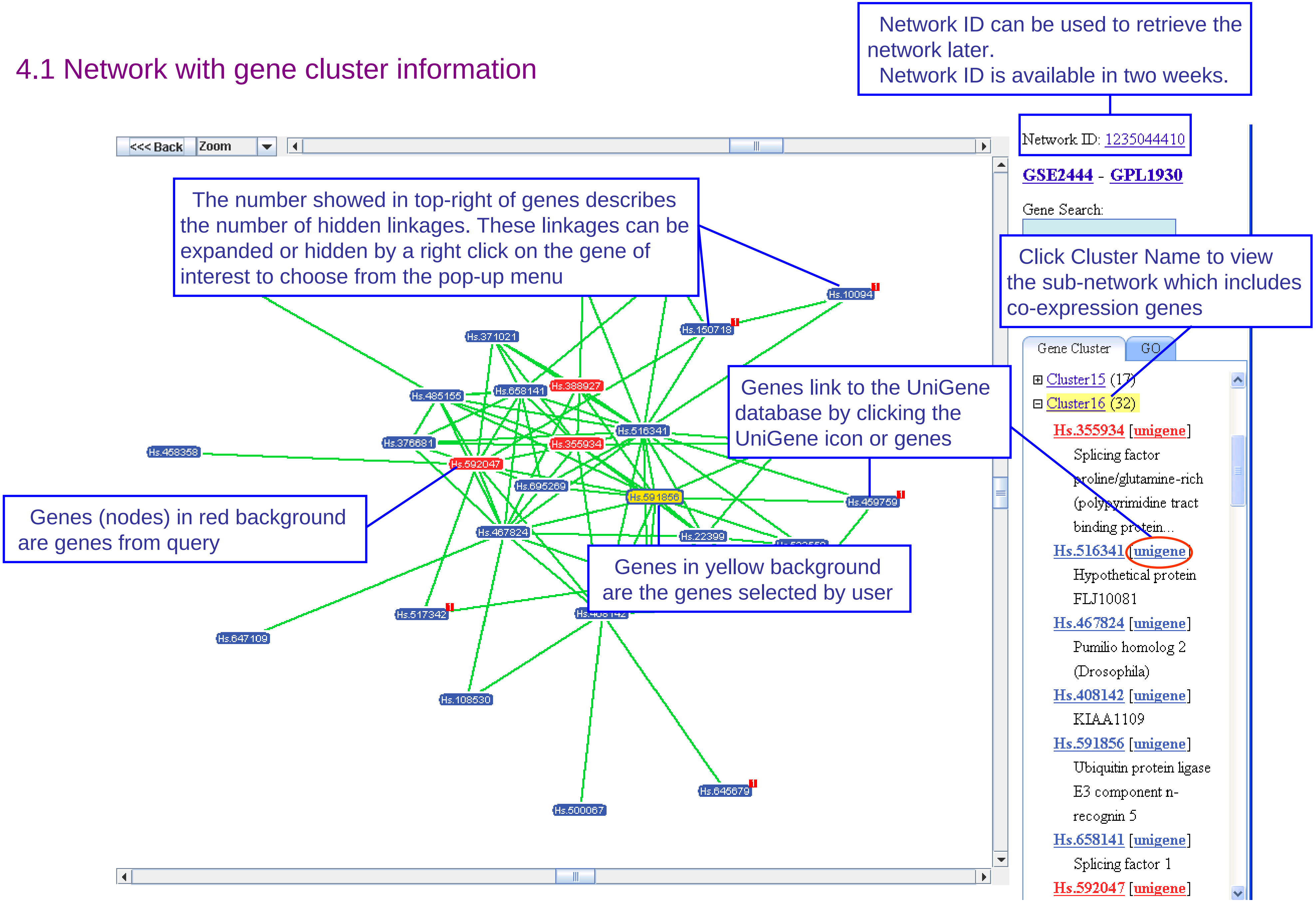

4.2 Network with GO annotation

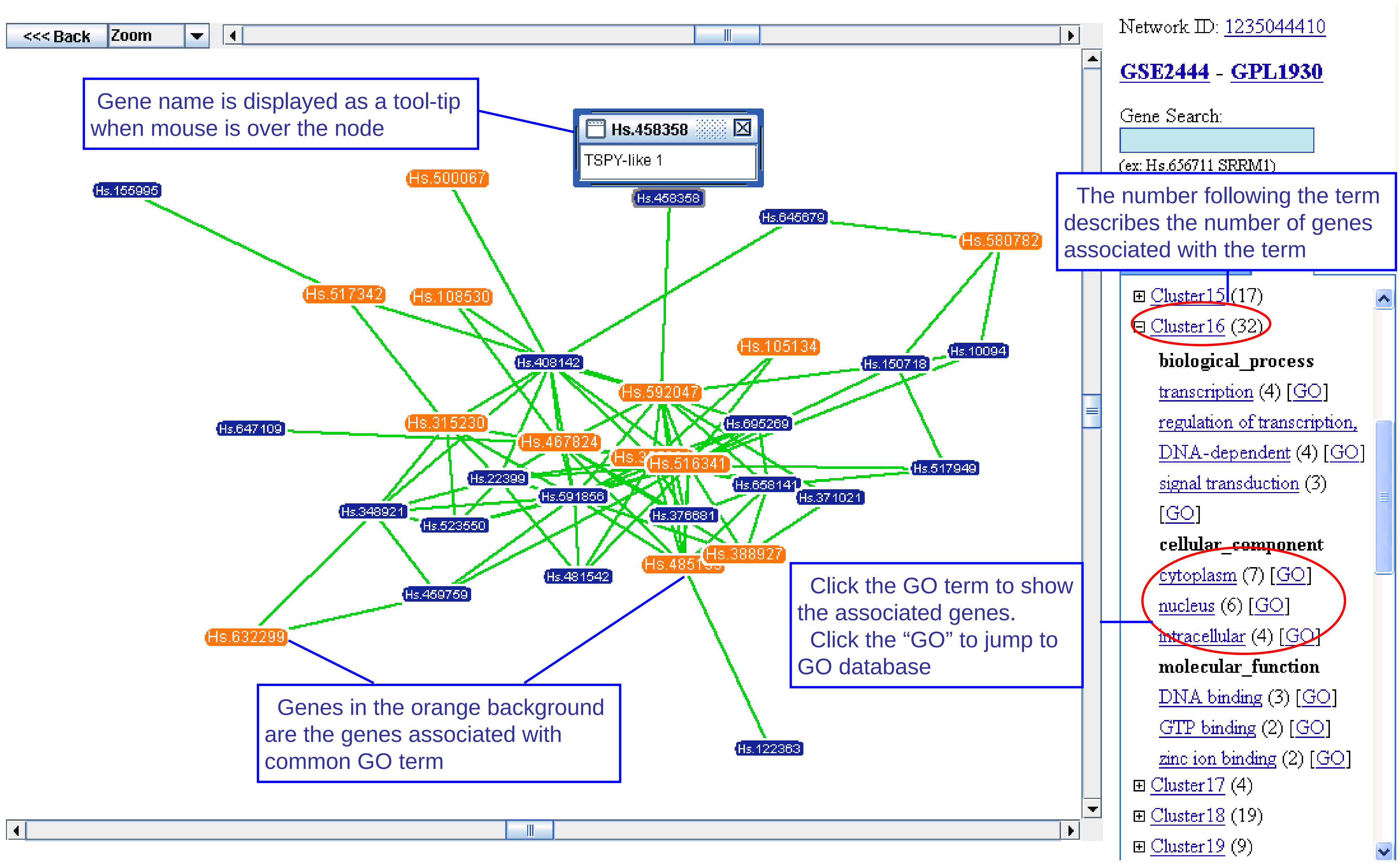

4.3 Gene search in the network

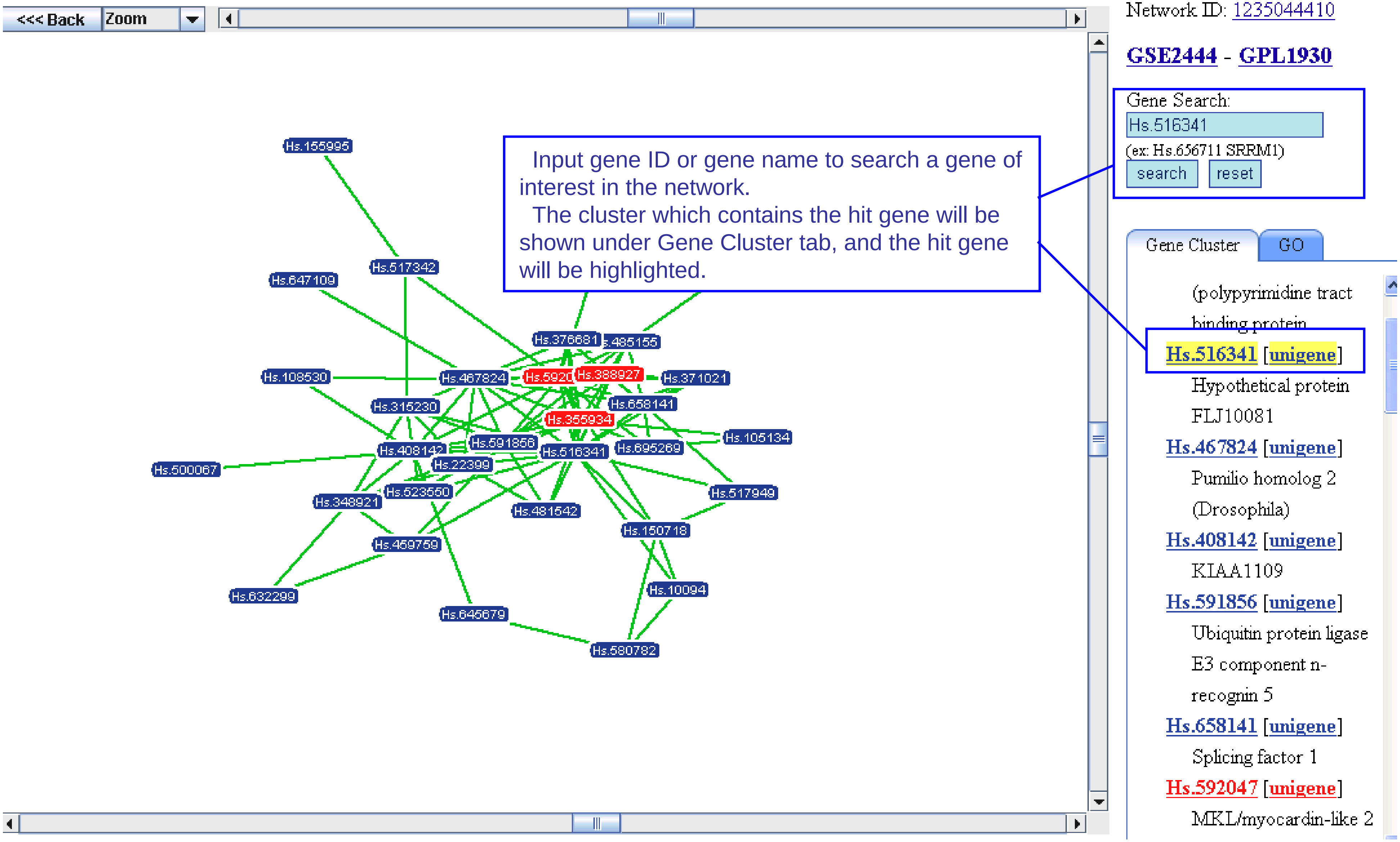

Supplement: Additional file 1 — User guide for GEM-TREND. User guide for GEM-TREND as a PDF file. [file 1471-2164-10-411-S1.pdf]
